# Supplementary material for: Restoration of the molecular clock is tumor suppressive in neuroblastoma
Source: Nat Commun. 2021 Jun 28;12:4006. doi: 10.1038/s41467-021-24196-4 (PMC8238982; doi:10.1038/s41467-021-24196-4)
Supplement: Supplementary file 1 — Supplementary Information [file 41467_2021_24196_MOESM1_ESM.pdf]

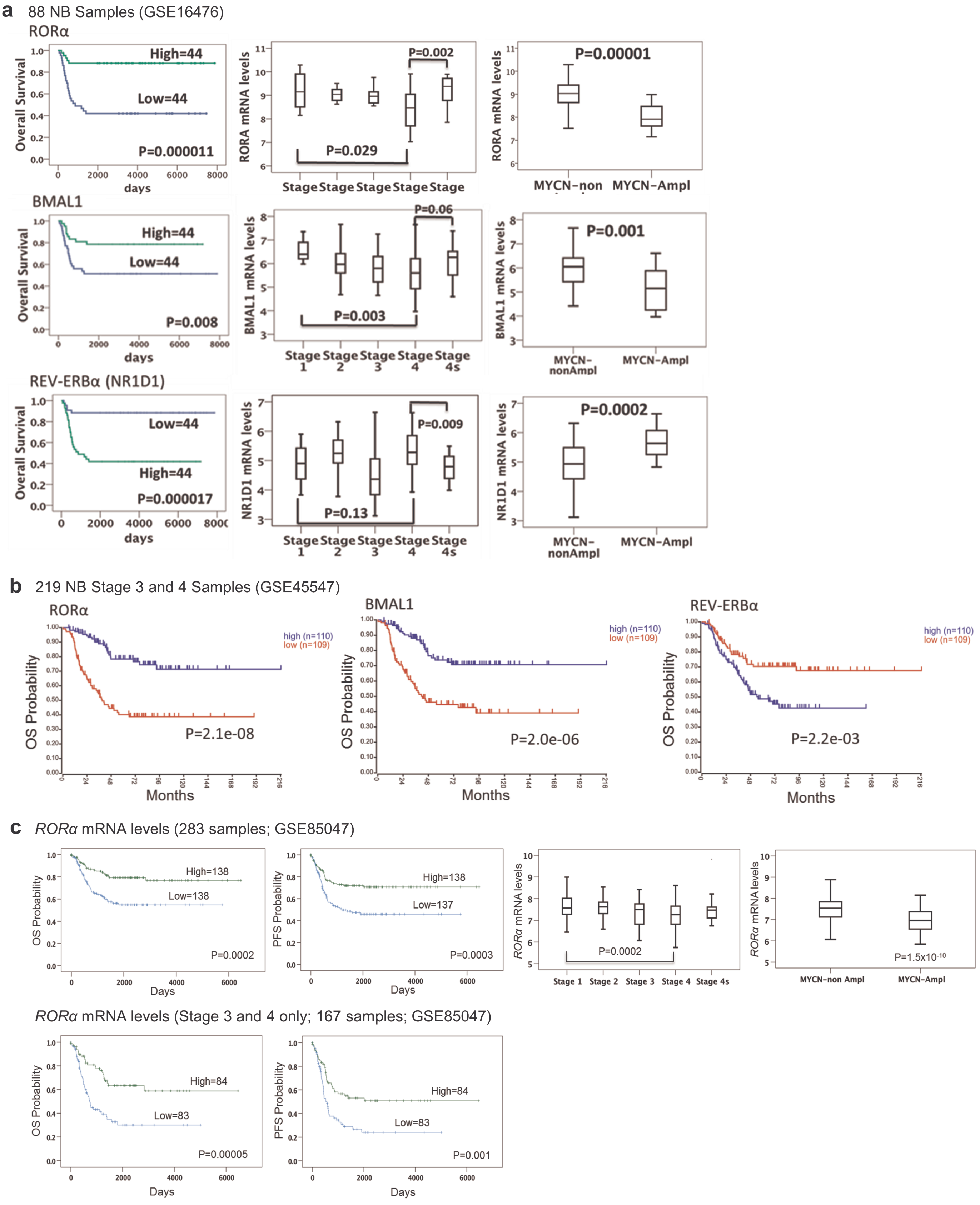

Supplementary Figure 1. Clock gene expression correlates with poor clinical outcome. a. Kaplan-Meier analysis of OS in patient cohort 2 (n=88, GEO: GSE16476). Graphs depict p-values corrected for multiple testing (Bonferroni correction) of cutoff levels for *RORα*, *BMAL1*, and *REV-ERBα*. Correlation between mRNA expression, INSS stages, and MYCN amplification is shown. **b.** Kaplan-Meier analysis of OS restricted to stage 3 and 4 in cohort 1 (GEO: GSE45547). **c.** Kaplan-Meier analysis of OS and PFS in patient cohort 3 (n=283, GEO: GSE85047) based on *RORα* mRNA expression in all patients vs. stage 3 and 4 patients only. Correlations with INSS stages and MYCN amplification are also shown. For 1a and 1c: The box plot is defined by two lines at the 25th percentile and 75th percentile. A line is drawn inside the box at the 50th percentile.

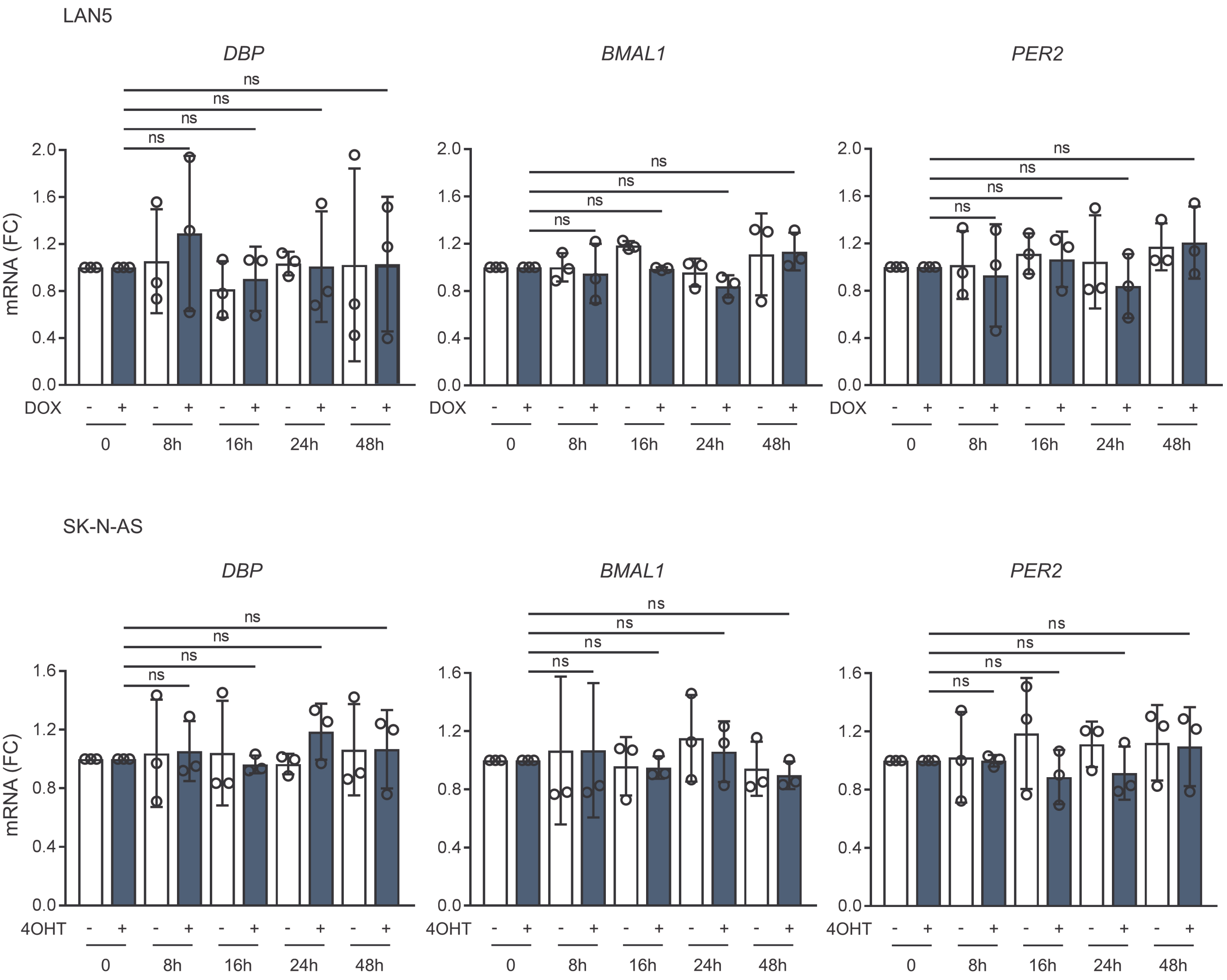

Supplementary Figure 2. Doxycycline and 4-OHT do not alter clock gene expression. mRNA expression levels of *DBP*, *BMAL1*, and *PER2* in MNA and non-MNA parental cells (LAN5 and SK-N-AS) upon DOX (2µg/ml) or 4OHT (1µg/ml) treatment (0–48h). Data are mean ± SEM (n=3; two-way ANOVA with Sidak's multiple comparisons test; ns=non-significant). FC=Fold Change.

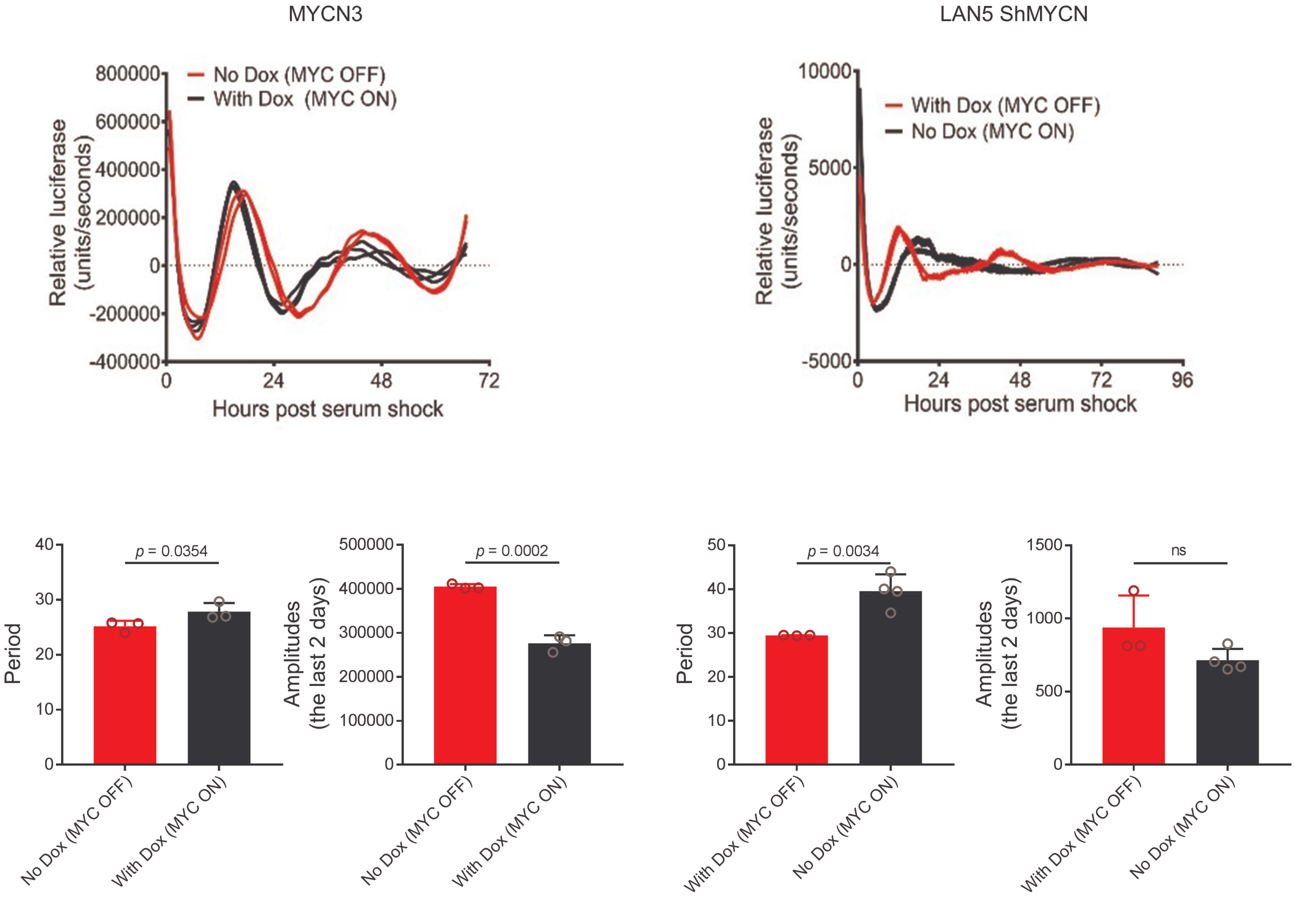

Supplementary Figure 3. *PER2* oscillation upon changes in MYCN expression. Real-time luminescence analysis: MYCN3 and LAN5 ShMYCN cells were treated with or without DOX for 48h before infection with *Per2*-luc adenovirus for 24h. Representative *PER2* oscillation and quantification of periods and amplitudes in the presence or absence of DOX are shown. Data are mean ± SD (n=3–4 biological replicates; one-tailed t-test; ns=non-significant).

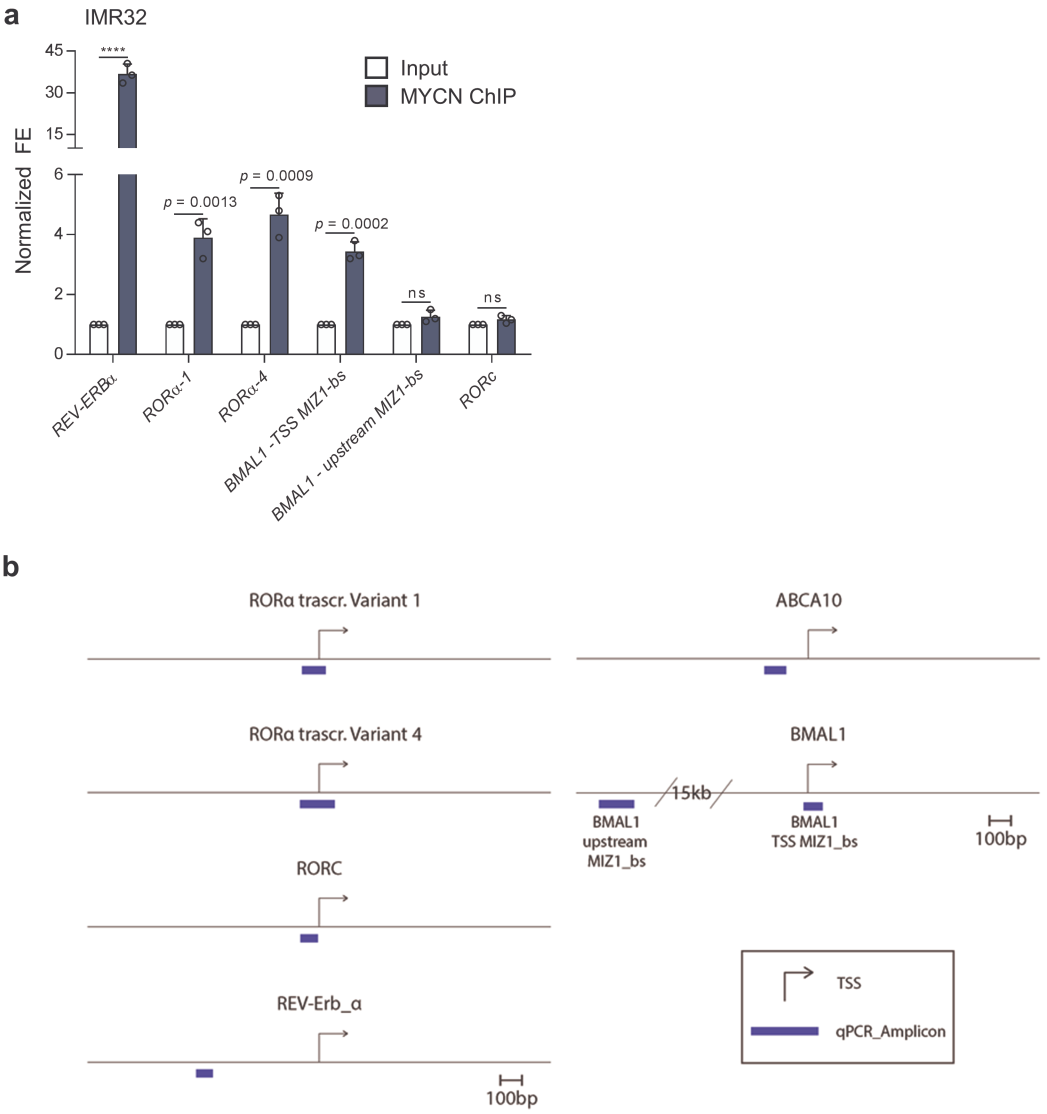

Supplementary Figure 4. MYCN binds to RORα, REV-ERBα, and BMAL1 gene promoters. a. MYCN ChIP-qPCR assays in IMR32 cells. Input (white bars) and MYCN IP (blue bars) samples were analyzed by q-PCR using specific primers for RORα (variants 1 and 4), REV-ERBα, BMAL1, and RORc (Supplementary Table 3). Data are mean ± SD (n=3 independent experiments; ****$p<0.0001$; two-tailed unpaired t-test). **b.** Graphical representation of the genomic regions analyzed in ChIP-qPCR assays.

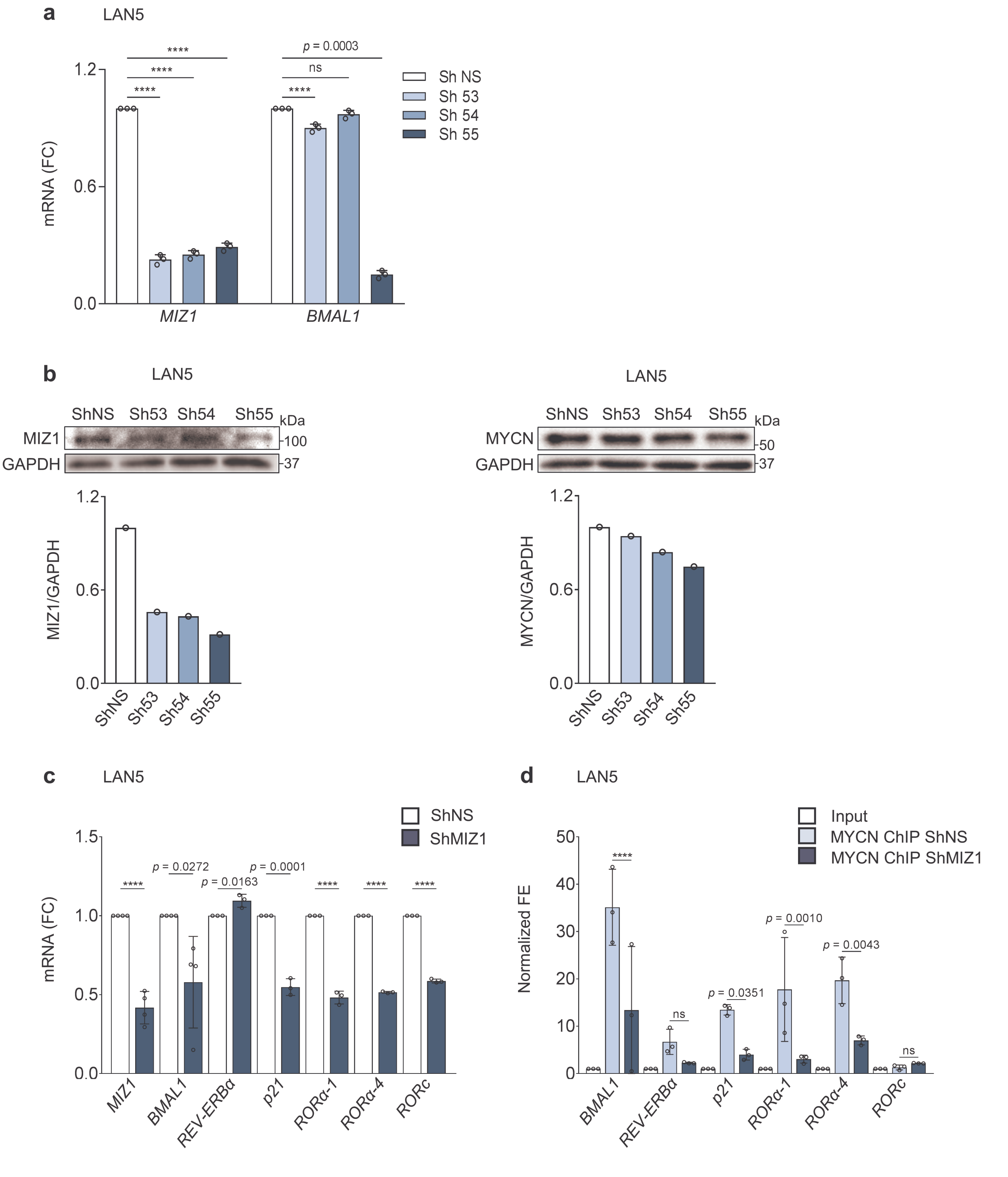

Supplementary Figure 5. MYCN repression of the clock requires MIZ1. a. *MIZ1* and *BMAL1* mRNA expression in LAN5 cells upon MIZ1 knockdown. Three independent ShMIZ1 sequences (Sh53, 54, and 55) were tested and samples were collected 48h post-transduction. Data are mean ± SD and are normalized to *GUSB* expression (n=3; ****p<0.0001; one-way ANOVA with Dunnett's multiple comparisons test). **b.** MIZ1 and MYCN protein expression upon MIZ1 knockdown (48h). GAPDH was used as loading control and MIZ1, MYCN/GAPDH ratios were determined (n=2). Data were analyzed by densitometry (ImageJ v1.42q). **c.** mRNA expression of *MIZ1*, *BMAL1*, *REV-ERBα*, *p21*, *RORα-1*, *RORα-4* and *RORc* in LAN5 ShCTRL (ShNS) and LAN5 ShMIZ1 (Sh55) cells. Data are mean ± SEM and are normalized to *GUSB* expression (n=3–4; ****p< 0.0001; two-tailed unpaired t-test). **d.** MYCN ChIP-qPCR assay in LAN5 ShCTRL (ShNS) and LAN5 ShMIZ1 (Sh55) cells. Input (white bars) and MYCN IP (blue bars). Data are mean ± SD (n=3; ****p<0.0001; two-way ANOVA with Dunnett's multiple comparisons test). FC=Fold Change; FE=Fold Enrichment.

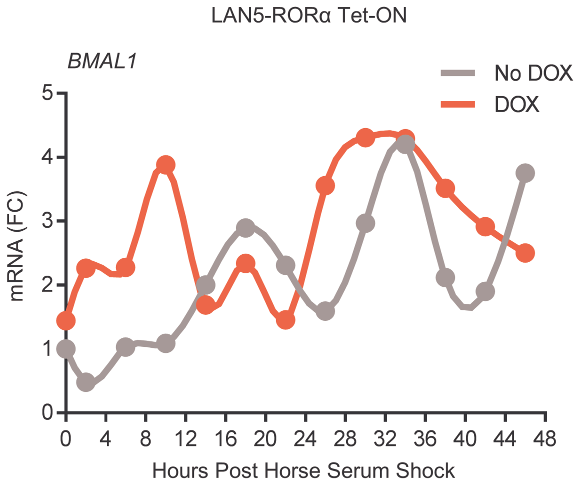

Supplementary Figure 6. *BMAL1* oscillatory activity in LAN5-RORα cells. LAN5-RORα cells were pre-cultured with (LAN5-RORα Tet-ON) or without (LAN5-RORα Tet-OFF) DOX for 48h before synchronization with 50% horse serum for 2h. Cell pellets were collected every 4h for 48h. *BMAL1* mRNA expression was determined by q-PCR and normalized to actin. Data are means from one biological replicate (n=2–4 technical replicates per time point). (FC)=Fold Change.

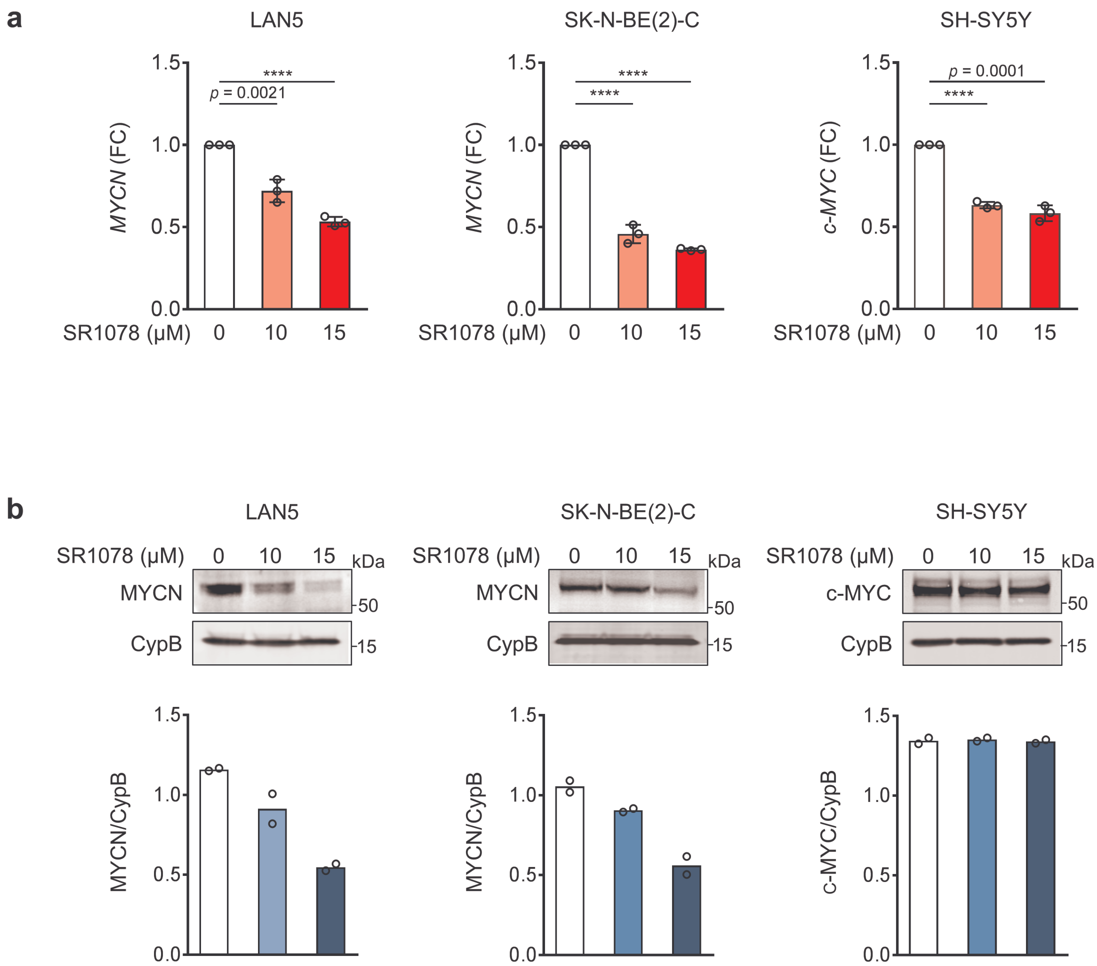

Supplementary Figure 7. Restoration of molecular clock is MYCN-dependent. mRNA (**a**) and protein expression (**b**) of MYCN and c-MYC in LAN5, SK-N-BE(2)-C (MNA), and SH-SY5Y (non-MNA) cells upon SR1078 treatment. mRNA data are mean ± SD (n=3 biological replicates; ****p<0.0001; two-tailed unpaired t-test). CypB served as protein loading control. Protein expression was analyzed by densitometry (ImageJ v1.42q) and MYCN/c-MYC/CypB ratios were determined (n=2). FC=Fold Change.

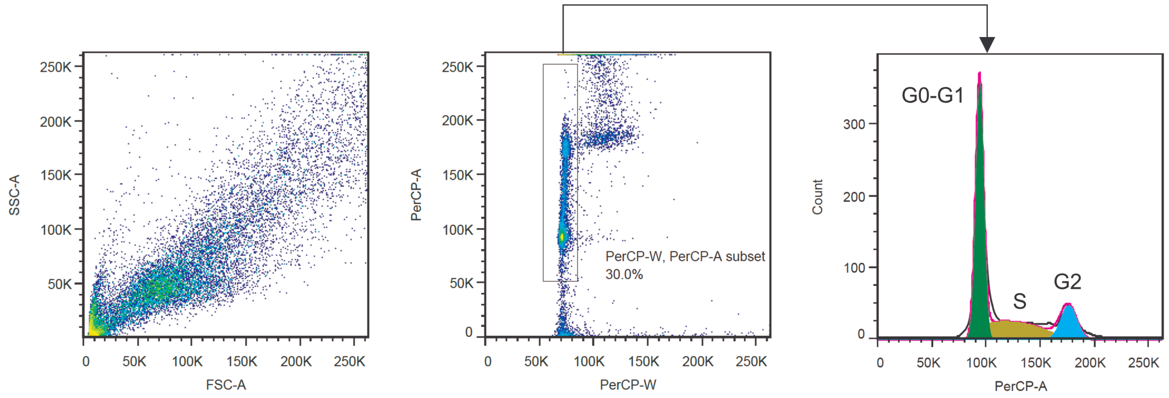

Supplementary Figure 8. Gating strategy for cell cycle analysis of LAN5 RORα cells. Cell cycle was analyzed by FlowJo v7.6.1. Single cells were gated on PerCP-W [(50-100)x1000]/PerCP-A[(50-250)x1000]. Histogram was presented as PerCP-A/Count. Percentage of cells in each phase was exported, and stacked bar chart was generated in GraphPad Prism v7.0 (corresponding to Figure 4B).

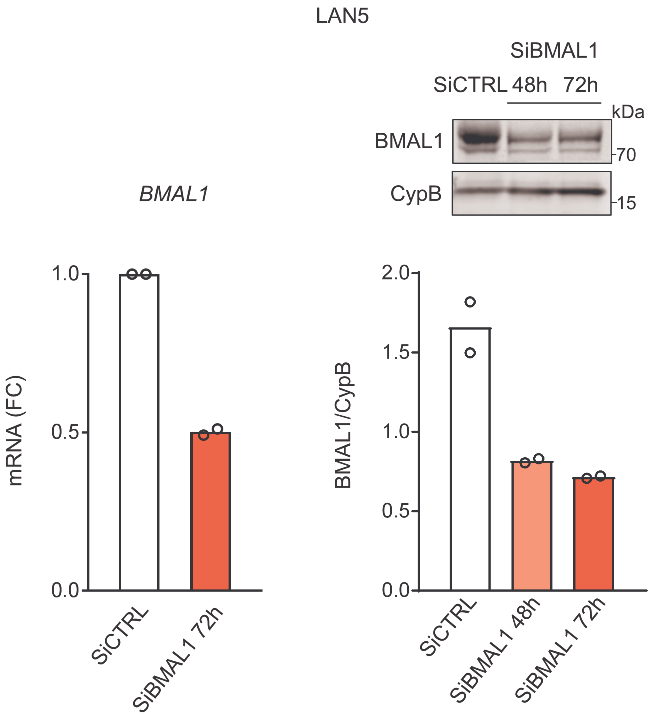

Supplementary Figure 9. BMAL1 mRNA and protein expression in LAN5 cells upon BMAL1 genetic knockdown. mRNA data are means (n=2 biological replicates; n=3 technical replicates). CypB served as protein loading control. Protein expression was analyzed by densitometry (ImageJ v1.42q) and BMAL1/CypB ratio was determined (n=2). (FC)=Fold Change.

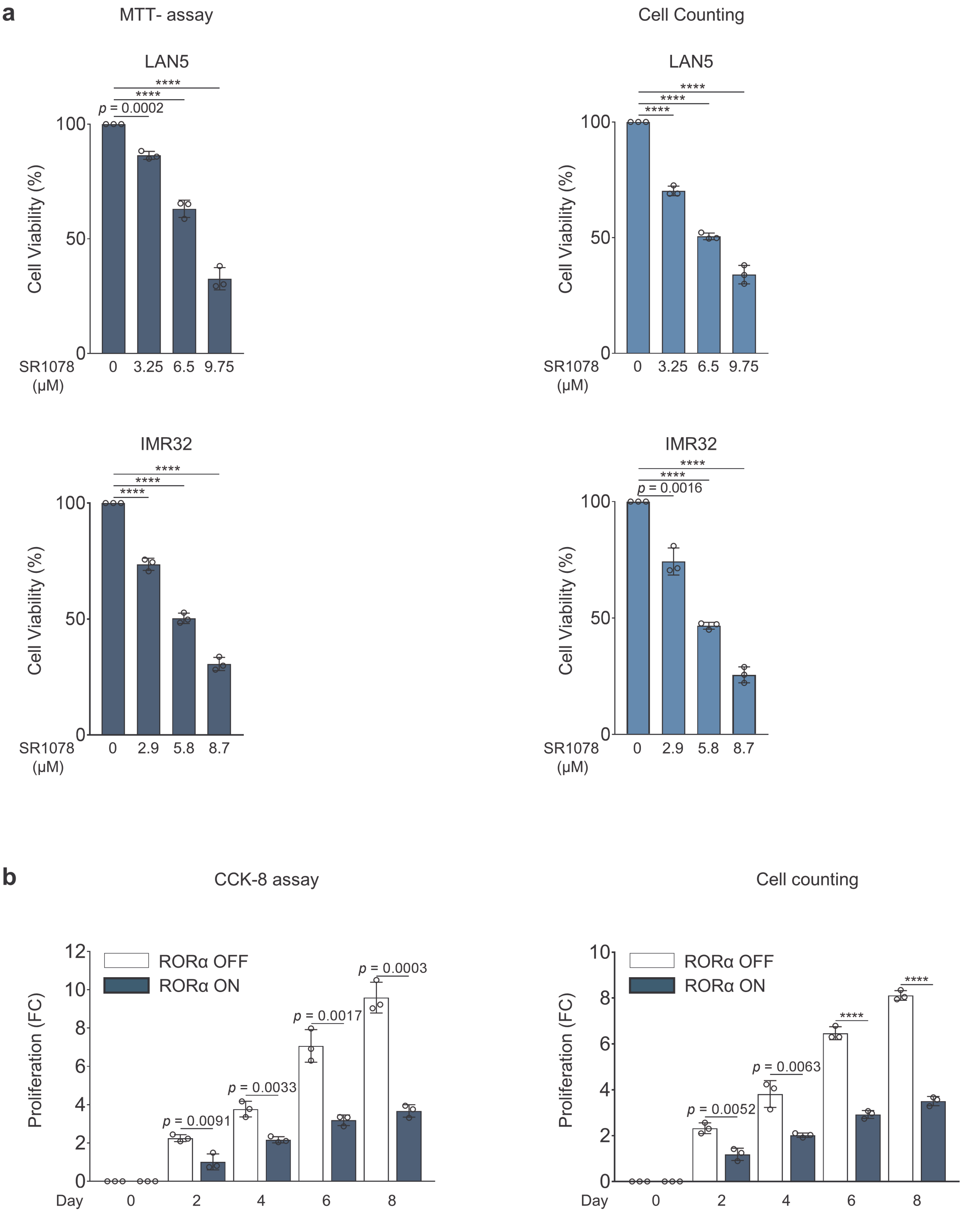

Supplementary Figure 10. Comparison of different cell proliferation assays. a. Cell viability of MNA LAN5 and IMR2 cells upon SR1078 treatment determined in parallel by MTT assay and cell counting (Neubauer chamber). Data are mean ± SD (n=3 biological replicates; ****p<0.0001, two-tailed unpaired t-test). **b.** Cell proliferation of LAN5-RORα Tet-ON/OFF cells by CCK-8 assay and cell counting (Neubauer chamber). Data are mean ± SD (n=3 biological replicates; ****p<0.0001, two-tailed unpaired t-test). (FC)=Fold Change.

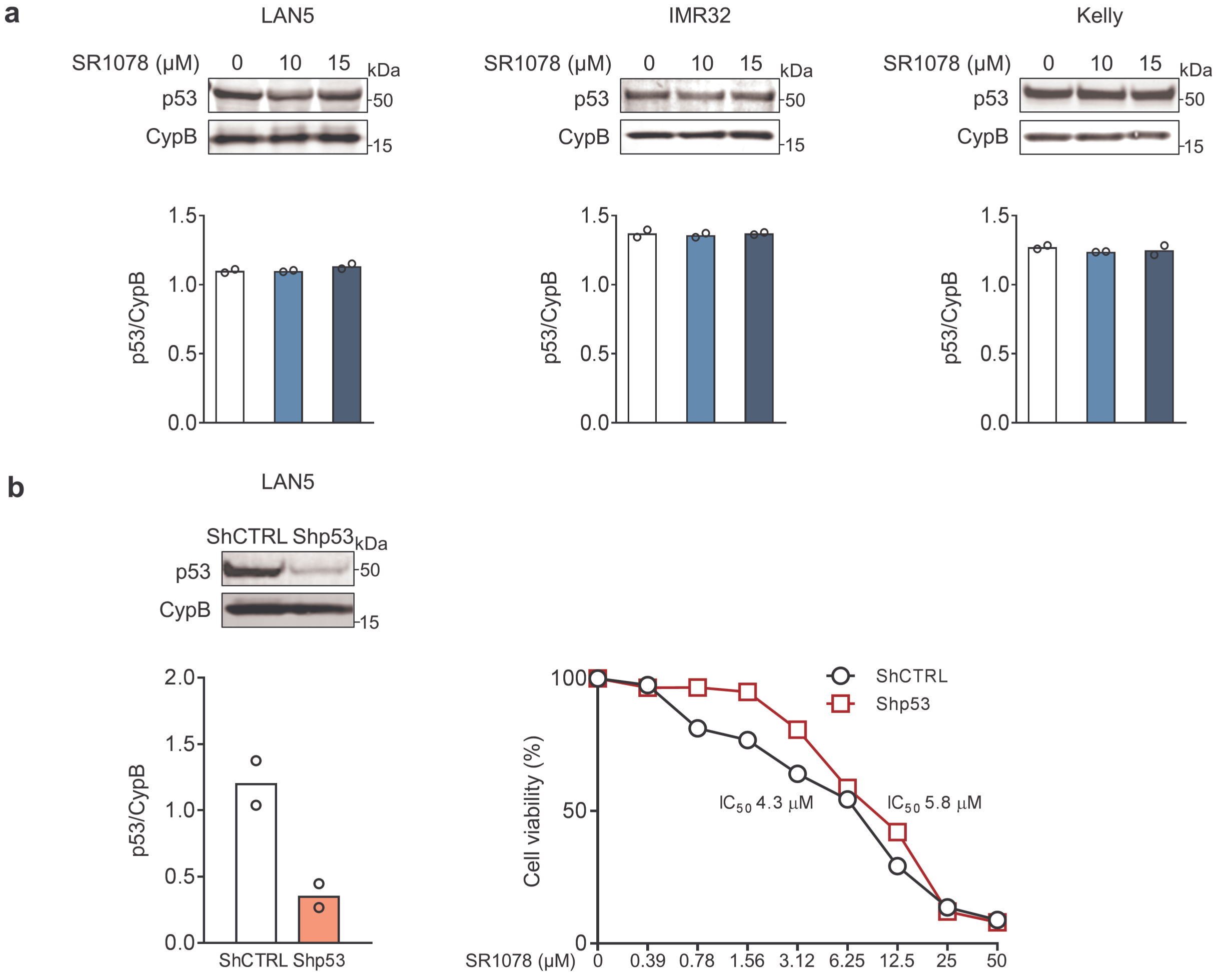

Supplementary Figure 11. SR1078 functions in a p53-independent manner. a. p53 protein levels in MNA LAN5, IMR32, and Kelly cells upon SR1078 treatment (24h). CypB served as loading control. Protein expression was analyzed by densitometry (ImageJ v1.42q) and BMAL1/CypB ratio was determined (n=2). **b.** p53 protein expression in LAN5 cells before (ShCTRL) and after (Shp53) genetic depletion of p53 (n=2). Cell viability (MTT assay) of LAN5 ShCTRL and Shp53 cells upon treatment with SR1078 for 72h. Data are means and are representative from n=2 biological replicates (n=4 technical replicates).

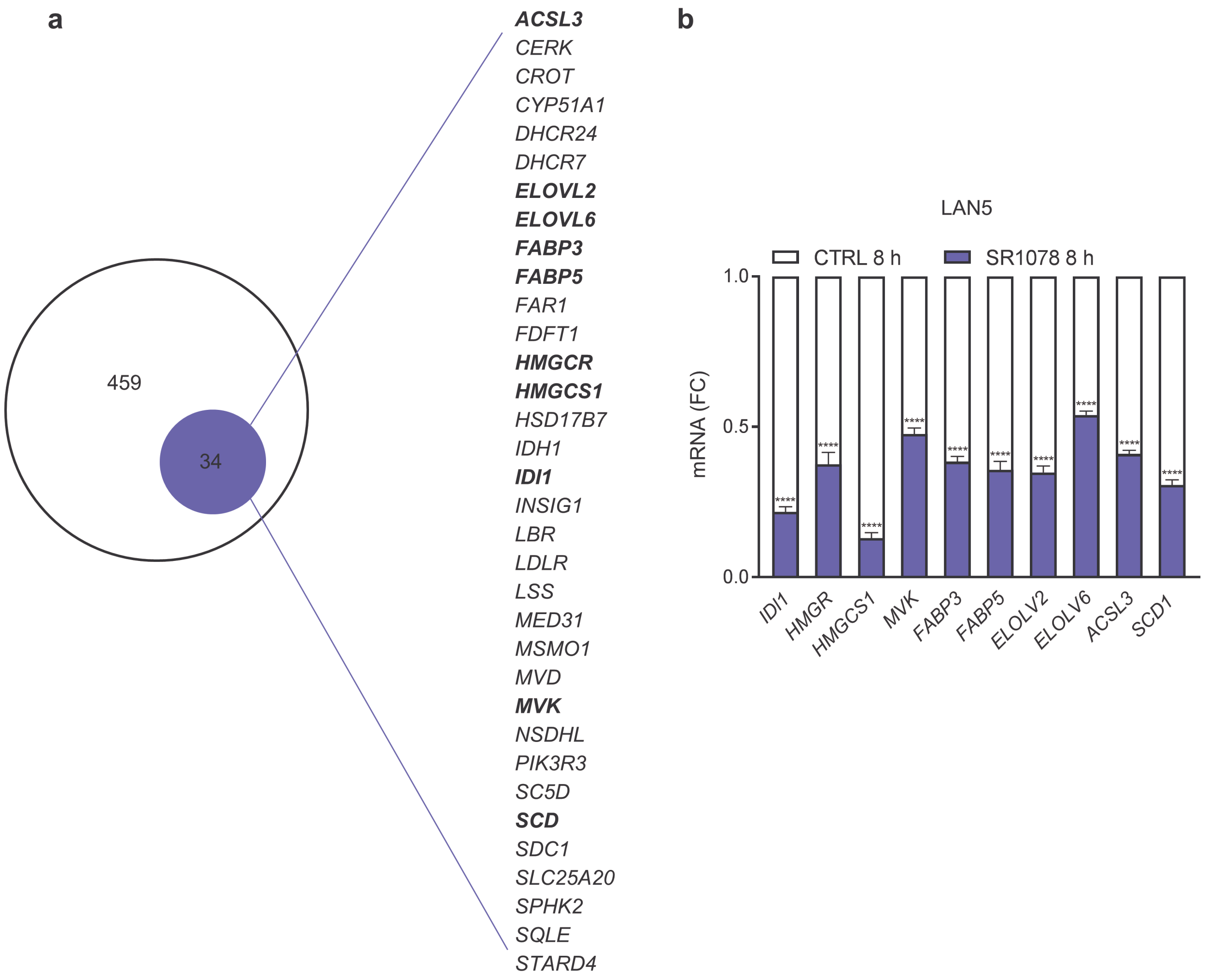

Supplementary Figure 12. SR1078-downregulated genes in LAN5 cells upon SR1078 treatment. a. Out of the 459 significantly downregulated genes, 34 genes belong to lipid metabolism pathways ($p<0.05$; two-tailed unpaired t-test; unadjusted p-values). **b.** mRNA expression of lipid metabolic genes (involved both in cholesterol and FA metabolism) in LAN5 cells upon SR1078 treatment (10μM for 8h). Data are mean ± SD (n=3; ****$p<0.0001$; two-tailed unpaired t-test). (FC)=Fold Change.

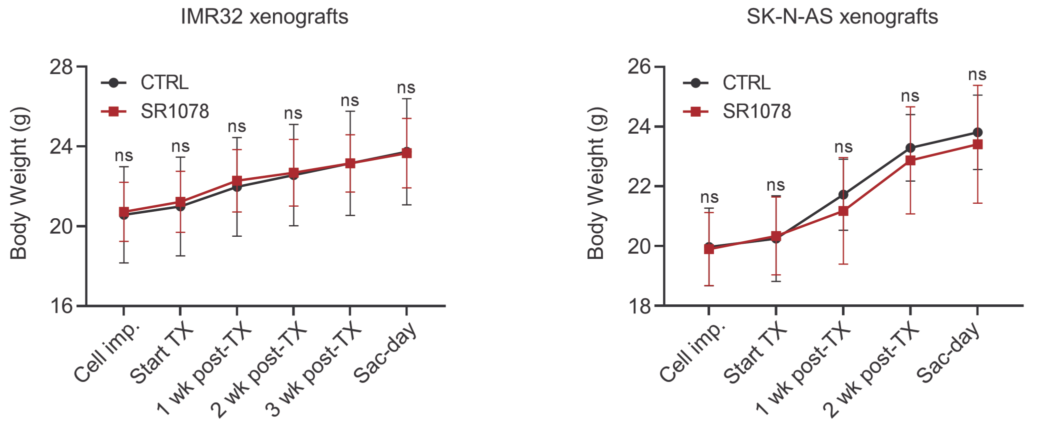

Supplementary Figure 13. SR1078 treatment does not affect mouse body weights. Body weights from MNA (IMR32) and non-MNA (SK-N-AS) engrafted mice (CTRL and SR1078 groups) recorded at different times throughout the study. Data are mean ± SD; two-way ANOVA with Sidak's multiple comparisons test; ns=non-significant.

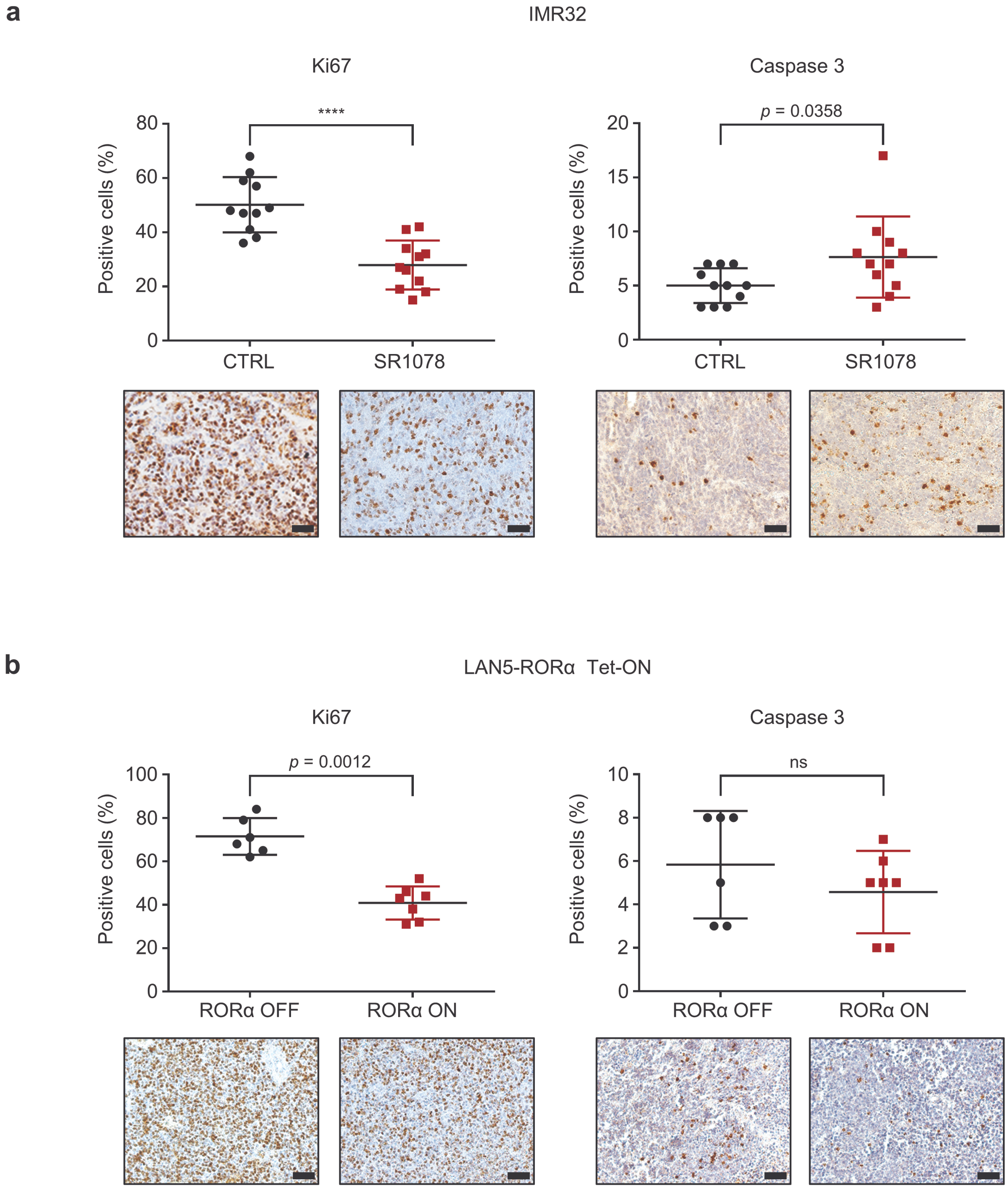

Supplementary Figure 14. **Anti-tumor activity of SR1078.** IHC analysis of tumor cell proliferation (Ki67) and apoptosis (cleaved caspase 3) in MNA xenografts treated with SR1078 (**a**) or overexpressing RORα (**b**). Four fields with 500 cells were evaluated by light microscopy, and percentages of Ki67 and caspase positive tumor cells were calculated. Values are mean ± SD (n=4). IMR32 xenografts: control group n=11; SR1078 group n=11(Ki67: p<0.0001; caspase: p=0.0358 by Mann-Whitney test); LAN5 RORα xenografts: RORα OFF group n=6; RORα ON n=7 (Ki67: p=0.0012; caspase: ns by Mann-Whitney test). Representative images (200x) with nuclear Ki67-positive cells and caspase positive cells are shown. Scale bar=50 μM; ns=non-significant.

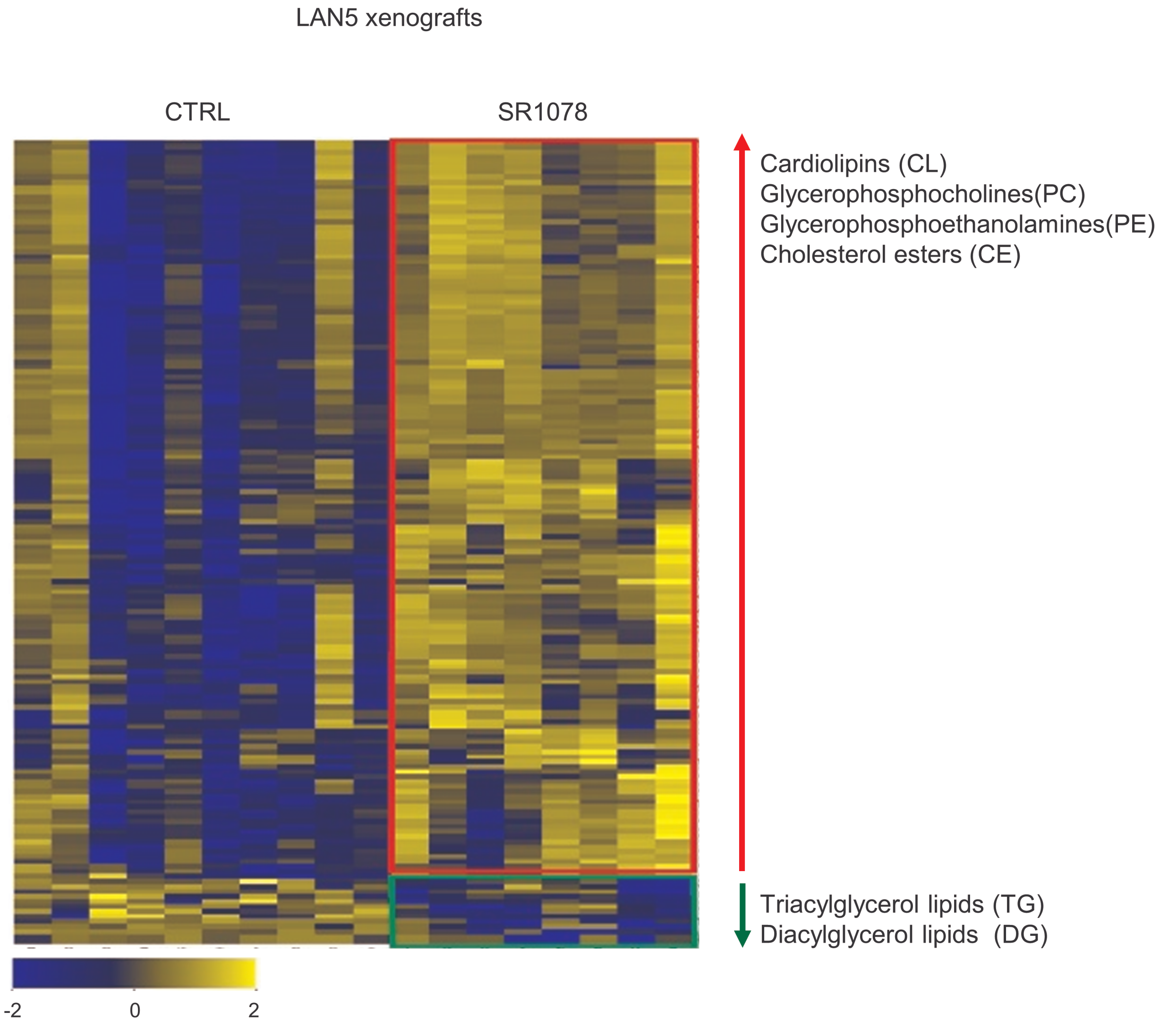

Supplementary Figure 15. Lipidomics analysis in LAN5 xenografts. Differentially altered lipids in LAN5-derived xenografts treated with vehicle control or SR1078 (15 mg/kg i.p. for 14 days). Groups were compared by two-tailed unpaired t-test. P-values were adjusted by the Benjamini-Hochberg procedure to obtain FDR. Changes with FDR<0.25 were selected for heat map presentation. Yellow=upregulated lipids; blue=downregulated lipids.

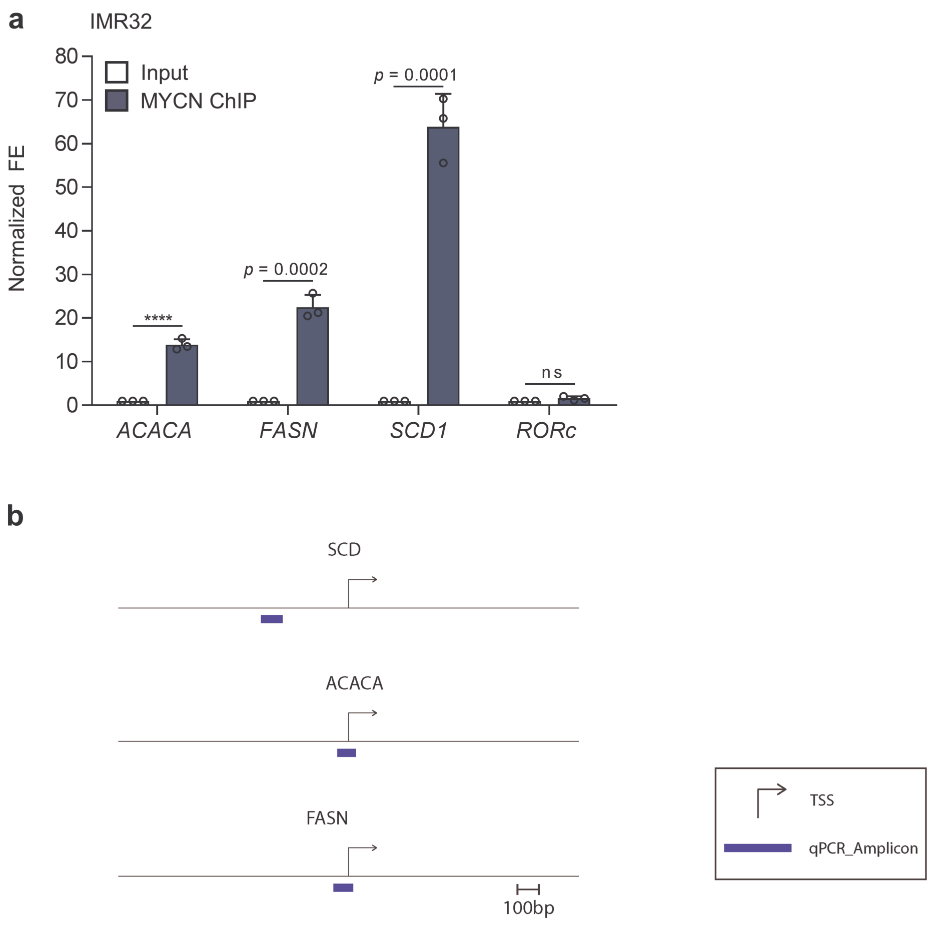

Supplementary Figure 16. MYCN binds to lipogenic gene promoters. a. MYCN ChIP in IMR32 cells (input=white bars; MYCN IP=blue bars). Samples were analyzed by q-PCR using specific primers for lipogenic enzymes *(ACACA*, *FASN, SCD1 and RORc* as negative control) (Supplementary Table 3). Data are mean ± SD (n=3 biological replicates; ****p<0.0001; two-tailed unpaired t-test; ns=non-significant). **b.** Graphical representation of the genomic regions analyzed in ChIP-qPCR assays. FC=Fold Change.

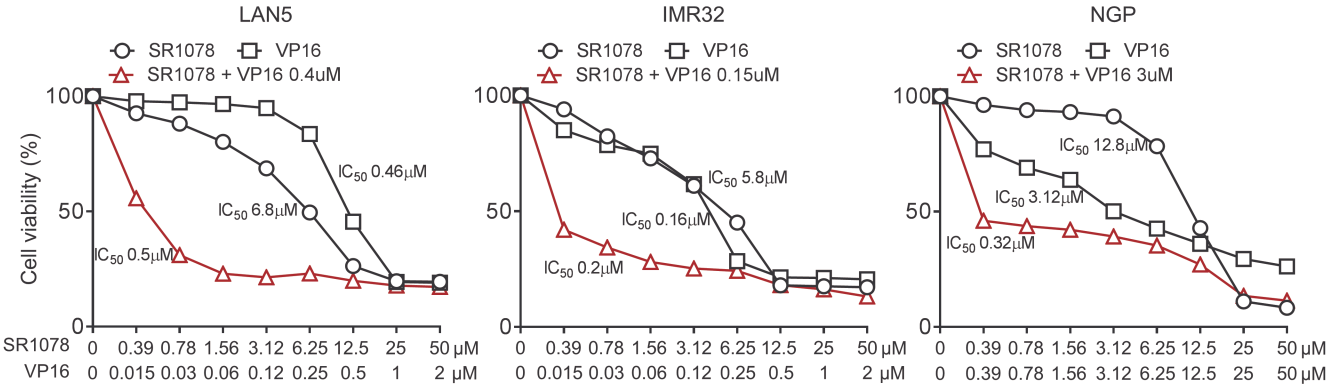

Supplementary Figure 17. VP16 sensitizes NB cells to SR1078. Cell viability (MTT assay) of MNA cells (LAN5, IMR32, and NGP) treated with SR1078, VP16, and their combination (IC_{50} values of VP16) for 72h. Data are means and are representative from n=2 biological replicates (n=3 technical replicates).

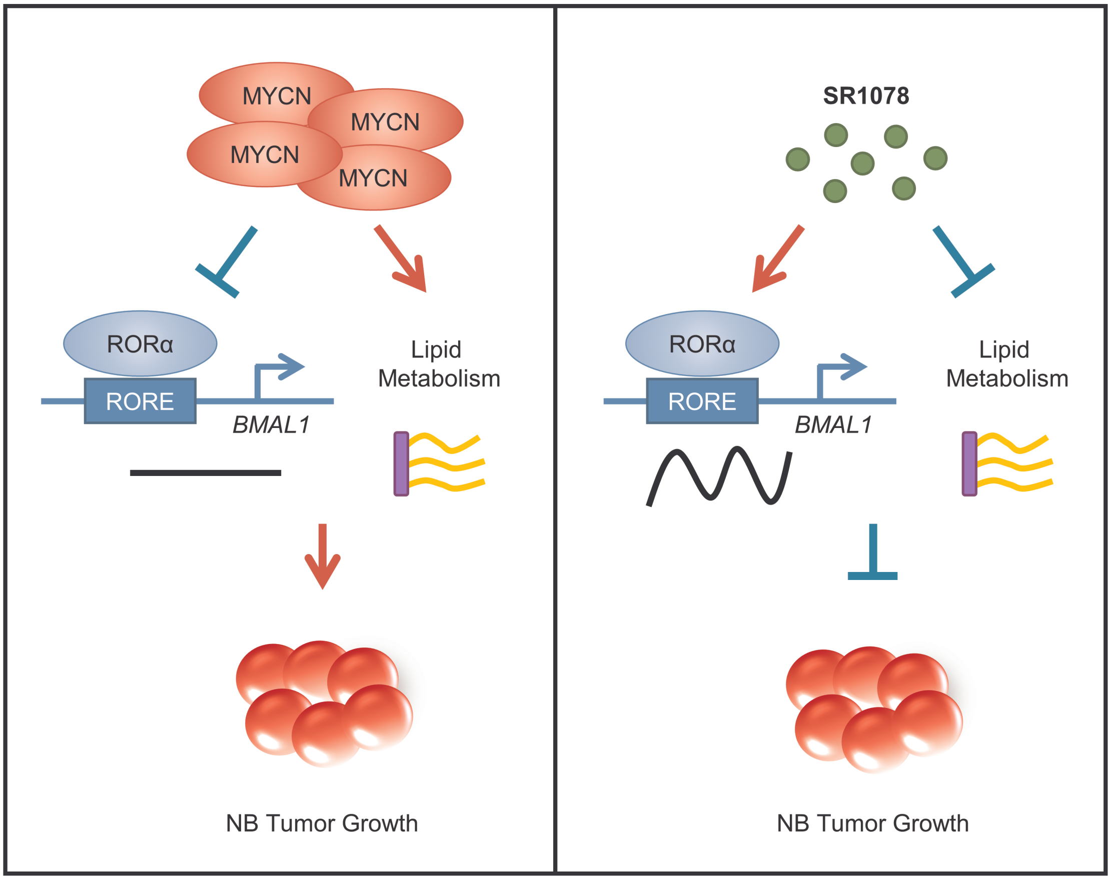

Supplementary Figure 18. Schematic representation of the regulation of the molecular clock and cell metabolism by MYCN and the ROR agonist SR1078.

Supplementary Table 1: *RORα* gene expression independently predicts clinical outcome

Multivariate Cox regression models for 476 NB based on OS and EFS considering prognostic markers and RORα

Markers	Overall Survival P-value	Overall Survival Hazard ratio	Overall Survival 95.0% CI Lower	Overall Survival 95.0% CI Upper	Event Free Survival P-value	Event Free Survival Hazard ratio	Event Free Survival 95.0% CI Lower	Event Free Survival 95.0% CI Upper
RORα (low vs. high)	1.00E-03	.35	.19	.67	1.40E-02	.61	.42	.91
Age at diagnosis (<18m vs. >=18m)	4.18E-04	3.00	1.63	5.51	8.00E-03	1.72	1.15	2.57
INSS Stage (1, 2, 3, 4s vs. 4)	9.00E-06	3.28	1.94	5.53	1.00E-05	2.31	1.59	3.33
MYCN (non-Ampl vs Ampl)	2.24E-07	3.29	2.09	5.16	3.00E-03	1.82	1.22	2.70

Multivariate Cox regression models for 88 NB based on Overall Survival considering prognostic markers and RORα

Markers	Overall Survival P-value	Overall Survival Hazard ratio	Overall Survival 95.0% CI Lower	Overall Survival 95.0% CI Upper
Single				
RORα (low vs. high)	**0.0001**	**0.15**	**0.06**	**0.40**
Age at diagnosis (<18m vs. >=18m)	0.00005	61.64	8.34	454.76
INSS Stage (1, 2, 4s vs. 3, 4)	0.001	29.11	3.96	214.12
MYCN (non-Ampl vs. Ampl)	1E-07	6.64	3.19	13.80
Multivariate				
RORα (low vs. high)	**0.03**	**0.33**	**0.12**	**0.88**
Age at diagnosis (<18m vs. >=18m)	0.0002	45.40	6.06	340.12
RORα (low vs. high)	**0.002**	**0.22**	**0.09**	**0.59**
INSS Stage (1, 2, 4s vs. 3, 4)	0.003	21.84	2.94	161.85
RORα (low vs. high)	**0.005**	**0.23**	**0.08**	**0.64**
MYCN (non-Ampl vs. Ampl)	0.001	3.87	1.78	8.41

Supplementary Table 1. *RORα* gene expression independently predicts clinical outcome. Multivariate Cox regression models based on OS and EFS of 476 (GEO: GSE45547, cohort 1) and 88 (GEO: GSE16476, cohort 2) patients considering available prognostic markers (age at diagnosis, INSS stage, and MYCN status) and ***RORα*** gene expression.

Supplementary Table 2: Analysis of intra-tumoral FAs in LAN5 xenograft tumors upon SR1078 treatment

Metabolite	CTRL	CTRL	CTRL	CTRL	CTRL	CTRL	CTRL	CTRL	SR1078	SR1078	SR1078	SR1078	SR1078	SR1078	SR1078
**Aminoheptanoic acid (AHA)**	-4.494292811	-4.527436431	-2.080674873	-3.162980018	-2.397673899	-3.785986599	-4.562554444	-0.681994615	-5.80965251	-3.926508555	-4.032378673	-4.378776189	-4.631142669	-4.065169408	-4.390378817
**Dodecanoic acid (DDA)**	1.406934296	1.607950756	2.179172626	2.037635426	1.999382701	1.938543007	1.928876418	2.34944494	1.672563002	2.064378692	1.76976449	1.440315237	1.545469442	1.948010197	1.607100721
**Tetradecanoic acid (TDA)**	1.017926788	-3.642065489	1.659367758	1.271420866	0.973624684	0.928655514	0.450078879	1.557469364	0.468213016	1.512252316	0.275881364	0.079046376	0.591312034	0.496017717	0.759382995
**Palmitoelic acid**	0.461741645	-0.87082086	0.631085506	0.602885716	0.530095643	0.572763564	0.366508521	0.147321593	0.152887692	0.581432566	-0.433677638	-0.131262529	-0.169004034	-0.133294052	0.356314009
**Eicosapentaenoic acid (EPA)**	5.938537439	5.914376413	5.168167613	6.504111793	5.435493305	6.531327113	5.684674756	5.091457392	5.849600276	6.038433087	6.755530695	6.025503288	6.577408195	5.931773045	6.000040803
**Eicosatrienoic acid (ETRA)**	0.33683513	-1.310959338	-0.992942444	0.225191726	0.013806566	0.168908158	-0.679719171	-0.274834679	-1.273211678	-1.625806502	-1.269026672	-1.145927256	-1.22568884	-1.634015744	-1.368251754
**OctadecEnoic acid (ODCA)**	-2.488069817	-1.676846722	-2.492390981	-2.788214932	-2.243593735	-2.134818878	-3.027401358	-3.077016403	-3.065080969	-3.663115849	-3.780705081	-3.636905132	-3.399359197	-3.125206991	-2.564515212
**Octadecadienoic acid (ODDA)**	1.541146385	1.202730887	2.187759913	1.770291524	1.419936321	1.576169831	1.793966736	2.293185656	1.798876186	2.459611573	1.676997371	1.350886226	2.300114372	1.102451316	1.215950782
**Hexadecanoic acid (HDA)**	3.500281883	3.522816586	4.246477599	3.946970515	4.002386194	3.937804382	3.696926062	4.279180499	3.455440439	3.947405837	3.865761532	3.353994288	3.833916864	3.096957997	3.686836604
**Docosahexaenoic acid (DHA)**	6.158508261	7.157763641	5.331252945	5.915111431	4.578125063	7.164930807	5.689581911	5.510297536	6.385033141	7.11188304	7.823460784	7.020581417	7.574647627	6.337649645	6.22265254
**Eicosatetraenoic acid (ETA)**	7.887036188	7.591653007	7.291996607	7.844186163	7.277209622	8.186760684	7.317346183	7.708945503	7.499280795	7.946336486	7.870946504	7.620502035	7.949027981	7.56547663	7.728617602
**Docosapentaenoic acid (DPA)**	4.466357784	3.468825105	3.024305108	4.468644612	3.174006835	4.784603146	3.522093101	3.242855064	3.821669577	4.139424988	3.675630046	3.470059916	3.4127277	3.874925282	4.381727586
**Docosatetraenoic acid (DTA)**	4.351376704	3.475776614	2.6405457	4.438151288	3.525766439	4.586879717	3.527903395	2.956733679	3.163155601	3.186284786	2.408188374	2.763831729	0.941387247	2.712586197	2.911687616
**OctadecAnoic acid (ODA)**	3.183398548	4.582048947	4.002105779	3.811985881	4.166723812	4.094524816	3.721050849	4.279552301	3.708971671	3.736875094	3.250301511	3.935638815	2.967491497	2.910594021	3.945568537

Method	Log Fold Change	Linear Fold Change	p-value_ttest	FDR
**Aminoheptanoic acid (AHA)**	-1.250301763	0.420360273	0.050632916	0.11814347
**Dodecanoic acid (DDA)**	-0.209906553	0.864593232	0.15306131	0.238095371
**Tetradecanoic acid (TDA)**	0.070383893	1.049996044	0.91861138	0.924587569
**Palmitoelic acid**	-0.27328395	0.827433945	0.24876346	0.348268843
**Eicosapentaenoic acid (EPA)**	0.384808828	1.305686758	0.135892448	0.237811784
**Eicosatrienoic acid (ETRA)**	-1.048918379	0.483330392	0.000842748	0.011798478
**OctadecEnoic acid (ODCA)**	-0.828225673	0.563221506	0.003737989	0.026165922
**Octadecadienoic acid (ODDA)**	-0.022450145	0.984559196	0.924587569	0.924587569
**Hexadecanoic acid (HDA)**	-0.285846385	0.820260246	0.09322271	0.18644542
**Docosahexaenoic acid (DHA)**	0.986933293	1.981967482	0.029486144	0.103201502
**Eicosatetraenoic acid (ETA)**	0.101885117	1.073174825	0.484256959	0.616327039
**Docosapentaenoic acid (DPA)**	0.056205098	1.039727235	0.849167667	0.924587569
**Docosatetraenoic acid (DTA)**	-1.104017185	0.465219287	0.012849789	0.059965681
**OctadecAnoic acid (ODA)**	-0.486539417	0.713735078	0.048002672	0.11814347

Supplementary Table 2. Analysis of intra-tumoral FAs in LAN5 xenograft tumors upon SR1078 treatment (CTRL tumors n=8 and SR1078 tumors n=7; FDR< 0.25). Data were normalized by median inter-quantile range normalization and were log_2 transformed. Groups were compared by two-tailed unpaired t-test; p-values were adjusted by the Benjamini-Hochberg procedure to obtain FDR.

Supplementary Table 3: Primers and SiBMAL1 target sequences

q-PCR primers		
Primer	Forward	Reverse
MYCN	5'CCGGGCATGATCTGCAA	5'CCGCCGAAGTAGAAGTCATCTT
c-MYC	5'CGTCTCCACACATCAGCACAA	5'CACTGTCCAACTTGACCCTCTTG
REV-ERBα	5'CCCCCTTGTACAGAATCGAAC	5'GAAGTTCGGTGATGGGGGA
RORα	5'AAACAAGCAGCGGGAGGTGA	5'TGGCAAACTCCACCACATAC
BMAL1	5'GAGAAGGTGGCCCAAAGAGG	5'GGAGGCGTACTCGTGATGTT
DKC1	5'GTTGACTACAGTGAGTCTGCCAA	5'TCACTCTCTCGCTTCCGCTT
FGF-21	5'ATCGCTCCACTTTGACCCTG	5'GGGCTTCGGACTGGTAAACA
G6PASE	5'TGAGGATGGAGGAAGGAA	5'GGGGAAGAGGACGTAGAA
FASN	5'ACAGGGACAACCTGGAGTTC	5'CTGTGGTCCCACTTGATGAGT
SCD1	5'TTCAGAAACACATGCTGATCCTCATAATTCCC	5'ATTAAGCACCACAGCATATCGCAAGAAAGT
ACACA	5'CCGCTTGCCTGACTTTTGAT	5'GCCTCAATTTCCCTTGCTGC
ODC1	5'CTTCGTGCAGGCAATCTCT	5'TCTCTTCAAATTTAAGTTTCACATCCT
IDI1	5'AATAAACACTAACCACCTCG	5'CTCGATGCAATAATCCTTTCTC
HMGR	5'ACTTCGTGTCATGACTTTC	5'GACATAATCATCTTGACCCTC
HMGCS1	5'TTGGCTTCATGATCTTTCAC	5'AATTTAACATCCCCAAAGGC
MVK	5'GAACTTGAGAACATTCCTCC	5'GATACCAATGTTGGGTAAGC
FABP3	5'CTTCAAGAACACAGAGATCAG	5'GAGTGTCAGGATGAGTTTTC
FABP5	5'5'AAGATGGGAAATTAGTGGTG	5'AACAGTATGGAGATTTGCTC
ELOLV2	AAGTTTCTTTGGACCAACAC	5'ACTTGTGCATAGATGGAAAC
ELOLV6	5'GGCAAAATGAGGAAAACAAC	5'CCTTGTGCCATTTTCTTTTG
ACSL3	5'AACCTCATAGCAAACCATTG	5'TCCTAGTTCTGGAATCCTTTC
PER2	5'GGGTGCGCTCGTTTGAACT	5'GGAACGAAGCTTTCGGACCTC
DBP	5'CGTGGAGGTGTTGATGACCTT	5'CGATGTCTTCGAGGGTCAAAG
18S	5'AAGTCCCTGCCCTTTGTACACA	5'GATCCGAGGGCCTCACTAAAC
GAPDH	5'TCACCACCATGGAGAAGGC	5'GCTAAGCAGTTGGTGGTGC
ChIP-qPCR primers:		
RORα trascr. Variant 4 FW	TGATGTCAGCCGGTCACATG	
RORα trascr. Variant 4 RV	CAGGGAGAGCGGATGGTC	
RORα trascr. Variant 1 FW	CTGCTTTTTCCCTCCTGCTTTC	
RORα trascr. Variant 1 RV	GTTTTCAGAGCAACTCTATGGTACC	
RORc FW	TCCACTGGTCTCTCCTCATGTC	
RORc RV	TCCAGCAGGCAGGGAAAAG	
REV-ERBα FW	TGTTCTCCCTAAGGCGAGTAGAAG	
REV-ERBα RV	TTCAGGGCAAAGTCCAGAGAAG	
BMAL1 TSS MIZ1-bs FW	GGATTGGTCGGAAAGTAGGTT	
BMAL1 TSS MIZ1-bs RV	CGGGTAAACAGGCACCTC	
BMAL1 upstream MIZ1_bs FW	TCCTGACTGCCACAGATCAA	
BMAL1 upstream MIZ1_bs FW	TCCCTCCTGTTCCAAGTGAG	
ABCA10 FW	AGCAACATCACCAACCTTATATTTCCC	
ABCA10 RV	TTAGTCAGTAAACACTCACTCAGTAAAGC	
SCD1 FW	CAGCCACCCCAGGATCTG	
SCD1 RV	CATTGTTCGCAGGCGTACC	
FASN FW	TTTGTCCGCACCACACCAG	
FASN RV	CAGGCCGCTGGAGCTC	
ACACA FW	GCCACCGCCCTCTTGG	
ACACA RV	CCCCATCCTCCCAGTCCTC	

SiBMAL1 target sequences:

1. CCAUUGAACAUCACGAGUA
2. CCACAUAGGUAUAGACAUG
3. GCACAUCGUGUUAUGAAUA
4. CGCGAUAGAUGGAAAGUUU

Supplementary Table 3. List of primer sequences and SiBMAL1 target sequences.
