# Supplementary material for: Restoration of the molecular clock is tumor suppressive in neuroblastoma
Source: Nat Commun. 2021 Jun 28;12:4006. doi: 10.1038/s41467-021-24196-4 (PMC8238982; doi:10.1038/s41467-021-24196-4)
Supplement: Supplementary file 3 — Description of Additional Supplementary Files [file 41467_2021_24196_MOESM3_ESM.pdf]

**Description of Additional Supplementary Files**

File Name: Supplementary Data 1
Description: **RNA-seq analysis in LAN5 cells upon SR1078 treatment** (8h, n=3 per group). Significantly up and downregulated genes are shown ($p<0.05$; two-tailed unpaired t-test; unadjusted p-values).

File Name: Supplementary Data 2
Description: **Lipidomics analysis in LAN5 xenograft tumors upon SR1078 treatment.** Control group tumors: n=10; SR1078 group tumors: n=8. Groups were compared by two-tailed unpaired t-test; p-values were adjusted by the Benjamini-Hochberg procedure to obtain FDR values.
